# Periodontal pathogenic bacteria, *Aggregatibacter actinomycetemcomitans* affect non-alcoholic fatty liver disease by altering gut microbiota and glucose metabolism

**DOI:** 10.1038/s41598-017-14260-9

**Published:** 2017-10-24

**Authors:** Rina Komazaki, Sayaka Katagiri, Hirokazu Takahashi, Shogo Maekawa, Takahiko Shiba, Yasuo Takeuchi, Yoichiro Kitajima, Anri Ohtsu, Sayuri Udagawa, Naoki Sasaki, Kazuki Watanabe, Noriko Sato, Naoyuki Miyasaka, Yuichiro Eguchi, Keizo Anzai, Yuichi Izumi

**Affiliations:** 1 Department of Periodontology, Graduate School of Medical and Dental Sciences, Tokyo Medical and Dental University (TMDU), Tokyo, Japan; 2 Division of Metabolism and Endocrinology, Faculty of Medicine, Saga University, Saga, Japan; 3 Eguchi Hospital, Ogi, Saga, Japan; 4 Department of Molecular Epidemiology, Medical Research Institute, Tokyo Medical and Dental University (TMDU), Tokyo, Japan; 5 Department of Comprehensive Reproductive Medicine, Tokyo Medical and Dental University (TMDU), Tokyo, Japan; 6 Liver Center, Saga University Hospital, Saga, Japan

## Abstract

Increasing evidence indicates that periodontitis affects non-alcoholic fatty liver disease (NAFLD). We examined the relationship between periodontal bacterial infection and clinical/biochemical parameters in 52 NAFLD patients. Anti-*Aggregatibacter actinomycetemcomitans* (*Aa*) antibody titers correlated positively with visceral fat, fasting plasma insulin, and HOMA-IR; and negatively with the liver/spleen ratio. C57BL/6J mice (8-weeks-old) were given *Aa* or saline (control) for 6 weeks, and were fed either normal chow (NCAa, NCco) or high-fat diet (HFAa and HFco). NCAa and HFAa mice presented impaired glucose tolerance and insulin resistance compared to control mice. HFAa mice showed higher hepatic steatosis than HFco animals. Liver microarray analysis revealed that 266 genes were differentially expressed between NCAa and NCco mice. Upregulated genes in *Aa*-administrated mice were enriched for glucagon signaling pathway, adipocytokine signaling pathway and insulin resistance. Consistently, plasma glucagon concentration was higher in NCAa mice. In addition, Akt phosphorylation was lower in the liver of NCAa/HFAa than in NCco/HFco mice. Based on 16S rRNA sequencing, *Aa* administration changed composition of the gut microbiota. Metagenome prediction in gut microbiota showed upregulation of fatty acid biosynthesis and downregulation of fatty acid degradation in *Aa*-administered mice. Thus, infection with *Aa* affects NAFLD by altering the gut microbiota and glucose metabolism.

## Introduction

Non-alcoholic fatty liver disease (NAFLD) is the most common chronic liver disease. NAFLD includes a wide spectrum of conditions ranging from non-alcoholic fatty liver (NAFL) to non-alcoholic steatohepatitis (NASH). Generally, NAFL shows a non-progressive clinical course, whereas NASH is a more serious form of NAFLD and can progress to cirrhosis or hepatocellular carcinoma^1,2^. Many risk factors related to the development of NAFLD have been proposed, such as obesity, diabetes, and insulin resistance^3,4^.

Periodontal disease is an inflammatory disorder caused by pathogenic oral microorganisms that can lead to the destruction of alveolar bone and connective tissues around the teeth^5,6^. Periodontal bacteria present in dental plaque possess various virulence factors, such as lipopolysaccharide (LPS), fimbriae, and enzymes, which can trigger inflammation in periodontal tissues^7^. Considering that the bacterial flora of the oral cavity differs from that of the gut^8^, there is a possibility that swallowed bacteria could affect the composition of gut microbiome.

Given the infectious nature of periodontal disease, patients with the disease show elevated IgG antibody titers against periodontopathic bacteria. These antibody titers correlate with severity of periodontal disease^9^. Periodontal infection has long been associated with an increased risk of various diseases, such as atherosclerotic vascular disease^10^ or type 2 diabetes^11^. Recently, several studies have indicated that periodontitis might influence NAFLD^12,13^. In addition, gut microbiota appear to mediate development and progression of NAFLD^14–16^.

In the present study, we first examined the relationship between periodontal disease and NAFLD by measuring IgG antibody titers to periodontopathic bacteria in NAFLD patients. Based on the results, we then investigated the influence of *Aggregatibacter actinomycetemcomitans* infection on gut microbiota, glucose/lipid metabolism, and liver steatosis in mice.

## Results

### Correlation between IgG antibody titers against periodontal pathogens and clinical/biochemical parameters in NAFLD patients

**Figure 1:**
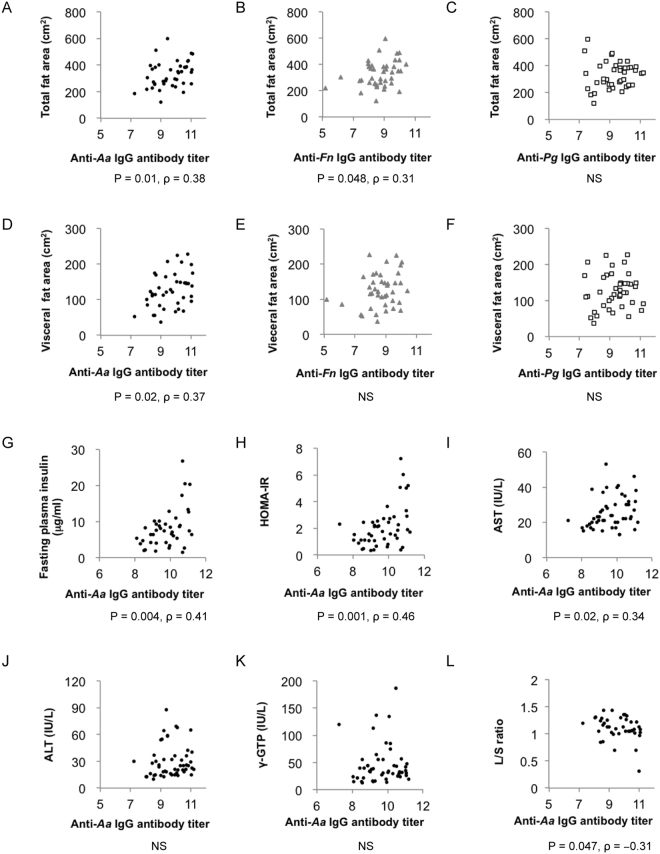
Correlations between IgG antibody titers to periodontal pathogen and clinical/biochemical parameters in NAFLD patients (n = 52). Correlation between total fat area and (**A**) anti-*Aa* IgG antibody titer, (**B**) anti-*Fn* IgG antibody titer, (**C**) anti-*Pg* IgG antibody titer. Correlation between visceral fat area and (**D**) anti-*Aa* IgG antibody titer, (**E**) anti-*Fn* IgG antibody titer, (**F**) anti-*Pg* IgG antibody titer. Correlation between anti-*Aa* IgG antibody titer and (**G**) fasting plasma insulin, (**H**) HOMA-IR, (**I**) AST, (**J**) ALT, (**K**) γ-GTP, (**L**) L/S ratio.

Clinical and biochemical characteristics of the subjects enrolled in this study are summarised in Table 1. We evaluated the correlation between IgG antibody titers to three major periodontopathic bacteria, *Aggregatibacter actinomycetemcomitans* ATCC 43718 (*Aa*), *Fusobacterium nucleatum* (*Fn*), *Porphyromonas gingivalis* ATCC 33277 (*Pg*), and clinical/biochemical parameters in NAFLD patients. Although there was no correlation between IgG antibody titers and body mass index (BMI), anti-*Aa* (P = 0.01, ρ = 0.38) and anti-*Fn* (P = 0.048, ρ = 0.31) antibody titers correlated significantly with total fat area evaluated by abdominal computed tomography (CT) scans (Fig. 1A and B). In contrast, no such correlation was observed for anti-*Pg* IgG antibody titer (Fig. 1C). Moreover, only anti-*Aa* IgG antibody titer showed a positive correlation with visceral fat area (Fig. 1D, P = 0.02, ρ = 0.37), whereas anti-*Fn* and anti-*Pg* titers did not (Fig. 1E and F). Anti-*Aa* IgG antibody titer correlated positively also with fasting plasma insulin (Fig. 1G, P = 0.004, ρ = 0.41) and the homeostasis model of assessment of insulin resistance (HOMA-IR) (Fig. 1H, P = 0.001, ρ = 0.46). A positive correlation was observed between anti-*Aa* IgG antibody titer and AST (Fig. 1I, P = 0.02, ρ = 0.34), but not ALT (Fig. 1J) or γ-GTP (Fig. 1K). Interestingly, anti-*Aa* IgG antibody titer showed a negative correlation with the liver/spleen (L/S) ratio (Fig. 1L, P = 0.047, ρ = −0.31). Correlations between anti-*Fn* IgG antibody titer/anti-*Pg* IgG antibody titer and several biochemical parameters are shown in Supplementary Figs S1 and S2.

**Table 1:** Characteristics of the patients with NAFLD.

Variables	All patients	Males	Females
	(n = 52)	(n = 27)	(n = 25)
Age (years)	55 ± 13.8	48 ± 14.6	63 ± 7.5
Weight (kg)	68 ± 12.0	75 ± 11.0	59 ± 6.2
BMI (kg/m^2^)	26 ± 2.8	27 ± 3.2	26 ± 2.2
Blood platelet count (×10^4^/μL)	22 ± 5.5	22 ± 5.3	22 ± 4.7
AST (IU/L)	26 ± 9.1	28 ± 9.5	23 ± 8.1
ALT (IU/L)	30 ± 18.2	37 ± 20.0	22 ± 12.2
ALP (IU/L)	243 ± 69.8	206 ± 63.2	284 ± 52.1
γ-GTP (IU/L)	57 ± 74.4	80 ± 95.8	31 ± 18.6
Fasting plasma glucose (mg/dL)	103 ± 19	106 ± 16	104 ± 11
HOMA-IR	2.1 ± 1.5	1.8 ± 1.1	2.4 ± 1.9
Total cholesterol (mg/dL)	219 ± 48	227 ± 46	218 ± 25
High density lipoprotein (mg/dL)	57 ± 14	54 ± 14	62 ± 12
Triglyceride (mg/dL)	145 ± 100	165 ± 128	124 ± 58
L/S ratio	1.00 ± 0.21	1.00 ± 0.26	1.00 ± 0.15

Data are shown as mean ± SD.

### *A*. *actinomycetemcomitans* administration causes increased body weight, impaired glucose tolerance, and insulin resistance

**Figure 2:**
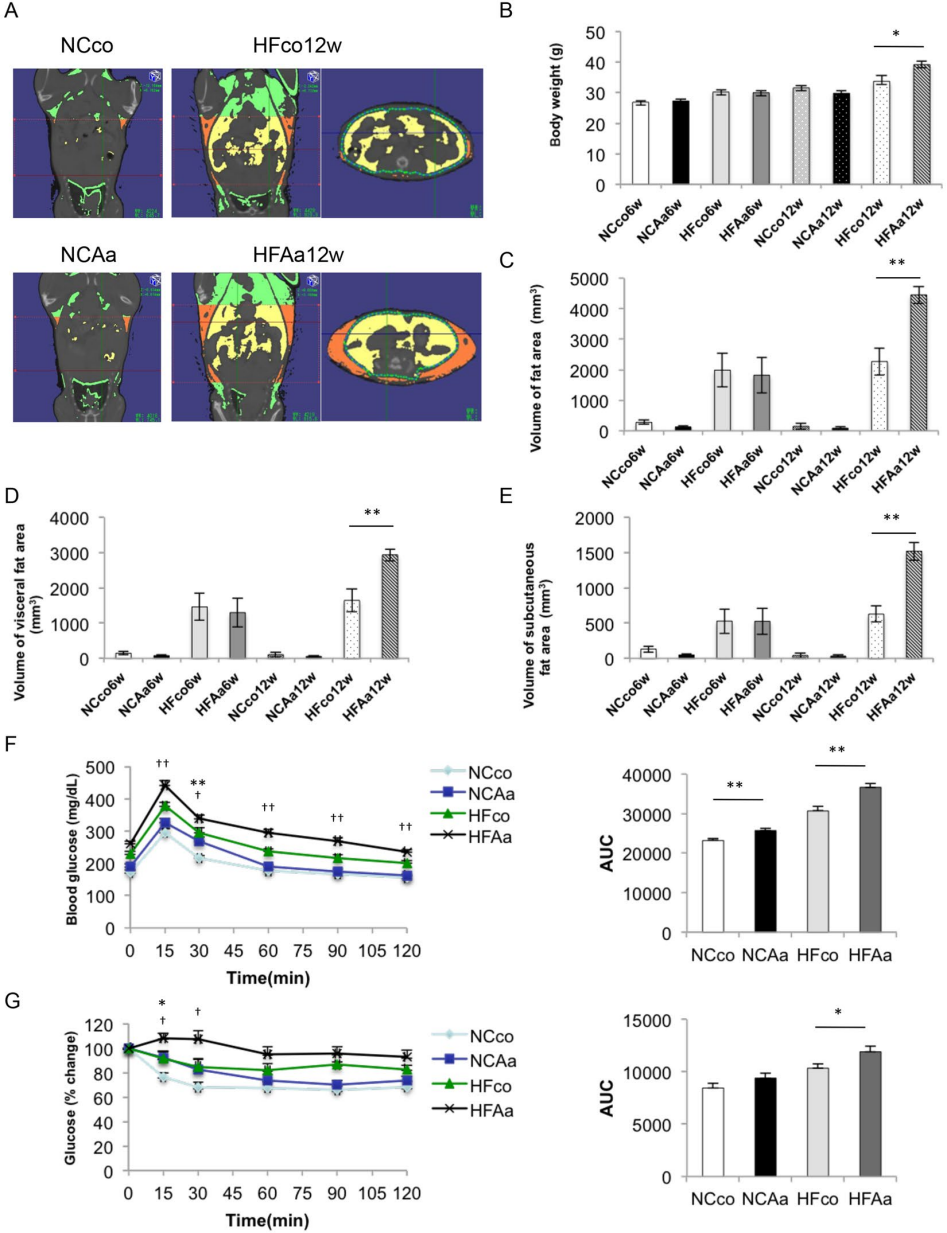
Comparison of body weight, body fat, glucose tolerance and insulin resistance among NCco, NCAa, HFco and HFAa mice. (**A**) Photographs of Micro-CT imaging. Yellow region represents visceral fat area and orange region represents subcutaneous fat area; (**B**) Body weight. (**C**) The volume of fat area, (**D**) the volume of visceral fat area and (**E**) the volume of subcutaneous fat area evaluated by Micro-CT imaging (n = 5). *P < 0.05, **P < 0.01. (**F**) OGTT (1 g/kg) and (**G**) ITT (1 U/kg) performed 6 h fasting at 6 weeks (n = 9–12). *P < 0.05, **P < 0.01 NCco vs NCAa, ^†^P < 0.05, ^††^P < 0.01 HFco vs HFAa.

Based on the significant correlation between *A*. *actinomycetemcomitans*, as opposed to other bacteria, and clinical/biochemical parameters of NAFLD patients, we conducted a 3D micro-CT analysis to quantify total and separated (visceral and subcutaneous) fat volumes (Fig. 2A). After 12 weeks, body weight was significantly higher in mice given a high-fat diet with *A*. *actinomycetemcomitans* administration (HFAa) than in high-fat diet control (HFco) animals. No significant differences could be detected between normal chow diet control (NCco) mice and normal chow diet with *A*. *actinomycetemcomitans* administration (NCAa) mice at 6 and 12 weeks, or between HFco and HFAa mice at 6 weeks (Fig. 2B). Total body fat (Fig. 2C), visceral fat (Fig. 2D), and subcutaneous fat (Fig. 2E) volumes were significantly higher in HFAa mice compared to HFco mice at 12 weeks. To determine whether administration of *A*. *actinomycetemcomitans* induced impaired glucose tolerance and insulin resistance, we performed a glucose tolerance test (GTT) (Fig. 2F) and an insulin tolerance test (ITT) (Fig. 2G) on both dietary groups at 6 weeks. Accordingly, administration of *A*. *actinomycetemcomitans* caused impaired glucose tolerance and insulin resistance in both dietary groups.

### *A*. *actinomycetemcomitans* administration increases liver steatosis

**Figure 3:**
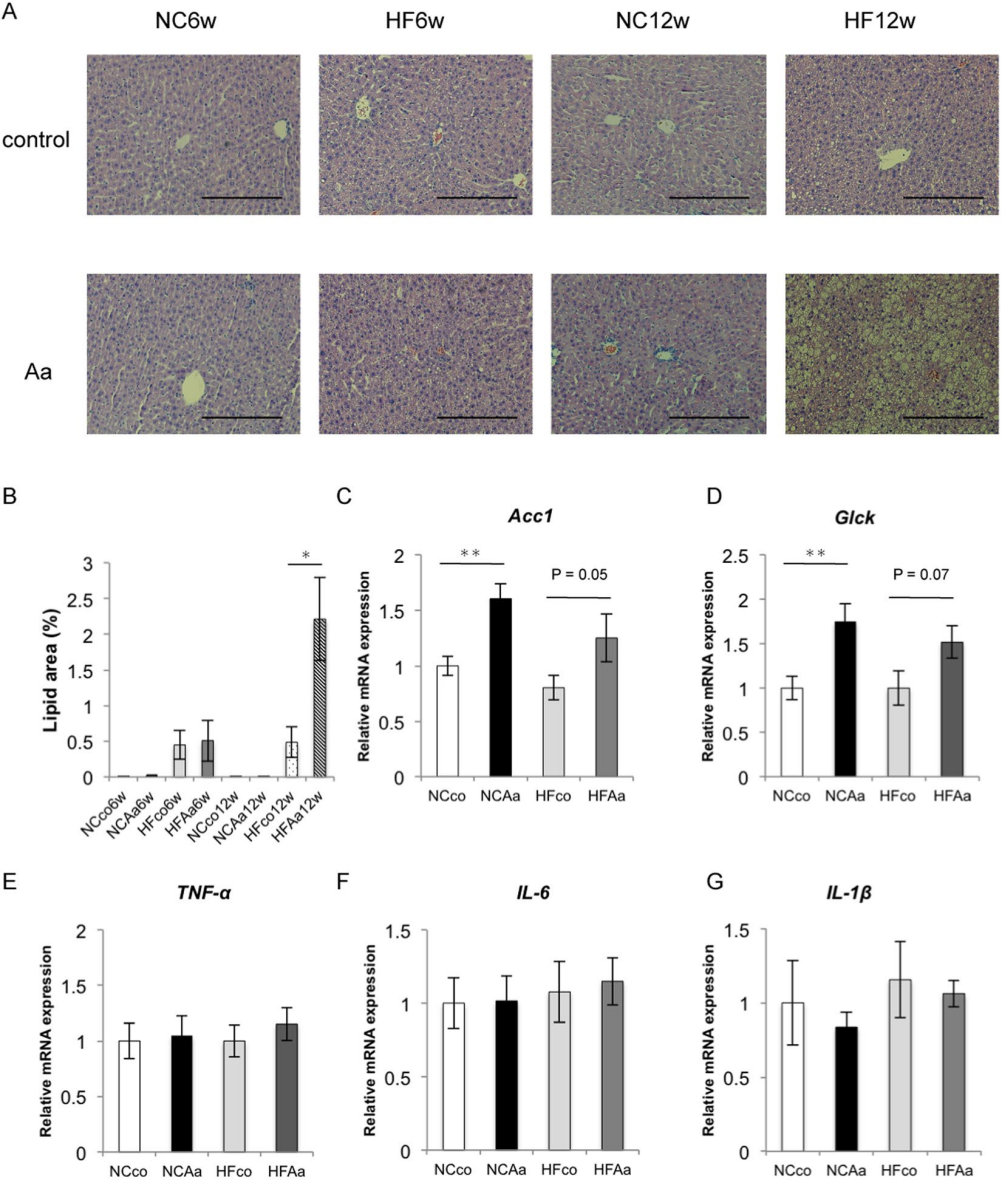
Evaluation of liver steatosis. (**A**) HE staining of liver tissue from NCco6w, NCAa6w, HFco6w, HFAa6w, NCco12w, NCAa12w, HFco12w and HFAa12w mice (row magnification × 200, Black bar = 100 μm), and (**B**) lipid area (%). (**C**) *Acc1* mRNA expressions; (**D**) *Glck* mRNA expressions; (**E**) *Tnfα* mRNA expressions; (**F**) *Il6* mRNA expressions; (**G**) *Il1β* mRNA expressions among NCco, NCAa, HFco and HFAa mice at 6 weeks (n = 9–12). *P < 0.05, **P < 0.01.

Histological analysis showed marked lipid accumulation in HFAa compared to HFco mice after 12 weeks (Fig. 3A and B). Oral administration of *A*. *actinomycetemcomitans* led to increased mRNA expression of Acetyl-CoA carboxylase (*Acc1*), an enzyme involved in lipid metabolism in the liver, and glucokinase (*Glck*) in mice fed a normal diet. *Acc1* and *Glck* mRNA expression in the liver of HFAa mice was also generally higher than in that of HFco mice (P = 0.05 and 0.07, respectively) as well as normal chow diet mice (Fig. 3C and D). Moreover, mRNA expression of tumour necrosis factor-alpha (*Tnfα*), interleukin (*Il*)-*6*, and *Il1β* in the liver did not differ significantly between NCco and NCAa mice, or HFco and HFAa mice at 6 weeks (Fig. 3E–G).

### Liver microarray analysis with or without *A*. *actinomycetemcomitans* administration

To obtain a comprehensive overview of gene expression in the liver following administration of *A*. *actinomycetemcomitans*, microarray analysis was performed in NCco and NCAa mice.

**Figure 4:**
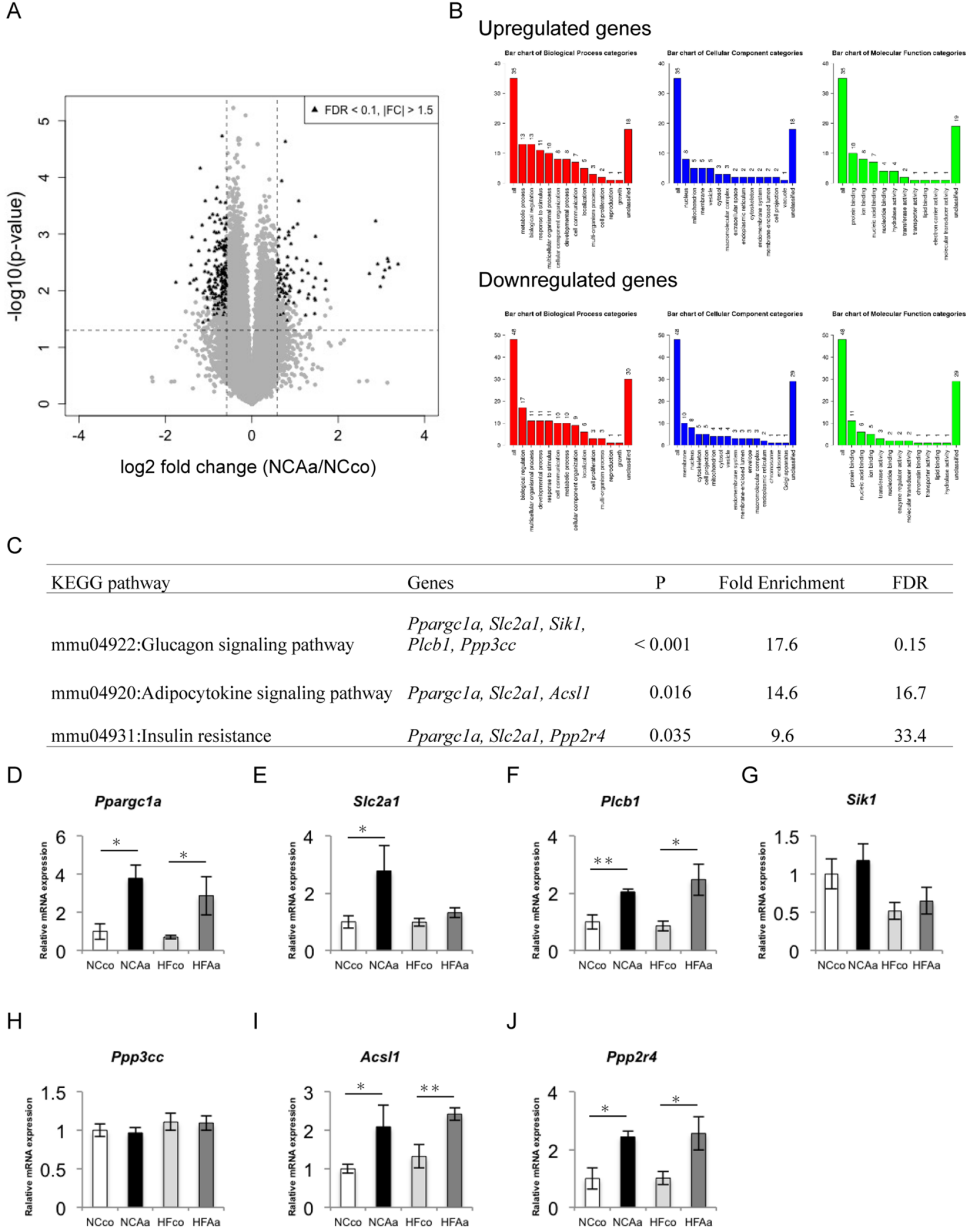
Microarray analysis in liver between NCco and NCAa mice (n = 4). (**A**) Volcano plots. (**B**) Gene Ontology in DEGs. (**C**) KEGG pathway (P < 0.05) in upregulated DEGs. Analysis of gene expressions in liver in (**D**) *Ppargc1a* mRNA expressions; (**E**) *Slc2a1* mRNA expressions; (**F**) *Plcb1* mRNA expressions; (**G**) *Sik1* mRNA; (**H**) *Ppp3cc* mRNA expressions; (**I**) *Acsl1* mRNA expressions; (**J**) *Ppp2r4* mRNA expressions among NCco, NCAa, HFco and HFAa mice at 6 weeks. *P < 0.05, **P < 0.01.

As shown in Fig. 4A, a total of 266 differentially expressed genes (DEGs) were identified, 71 of which were upregulated. All DEGs and corresponding Gene Ontology (GO) terms are listed in Supplementary Table S1, and those with higher expression (average normalised expression levels >first quantile) are clustered in the heatmap (Supplementary Fig. S3). Pathway analysis was used to uncover the significant pathways within differentially expressed gene sets according to the GO and Kyoto Encyclopedia of Genes and Genomes (KEGG) databases. Of the data sets with DEGs, 58 upregulated and 128 downregulated Entrez genes were identified in NCAa compared to NCco animals. GO slim overviewed the ontology content in upregulated and downregulated DEGs, respectively (Fig. 4B). Pathway analysis showed that the glucagon signaling pathway was significantly enriched in upregulated DEGs. In addition, adipocytokine signaling pathway and insulin resistance showed P < 0.05 (Fig. 4C). Quantitative PCR validated significant hepatic upregulation of peroxisome proliferative activated receptor, gamma, coactivator 1 mRNA (*Ppargc1a*), solute carrier family 2 (facilitated glucose transporter), member 1 mRNA (*Slc2a1*), phospholipase C, beta 1 mRNA (*Plcb1*), acyl-CoA synthetase long-chain family member 1 mRNA (*Acsl1*), and protein phosphatase 2A activator, regulatory subunit B mRNA (*Ppp2r4*) in animals treated with *A*. *actinomycetemcomitans* and fed a normal chow diet and/or high-fat diet (Fig. 4D–J).

### Glucose metabolism in the liver

**Figure 5:**
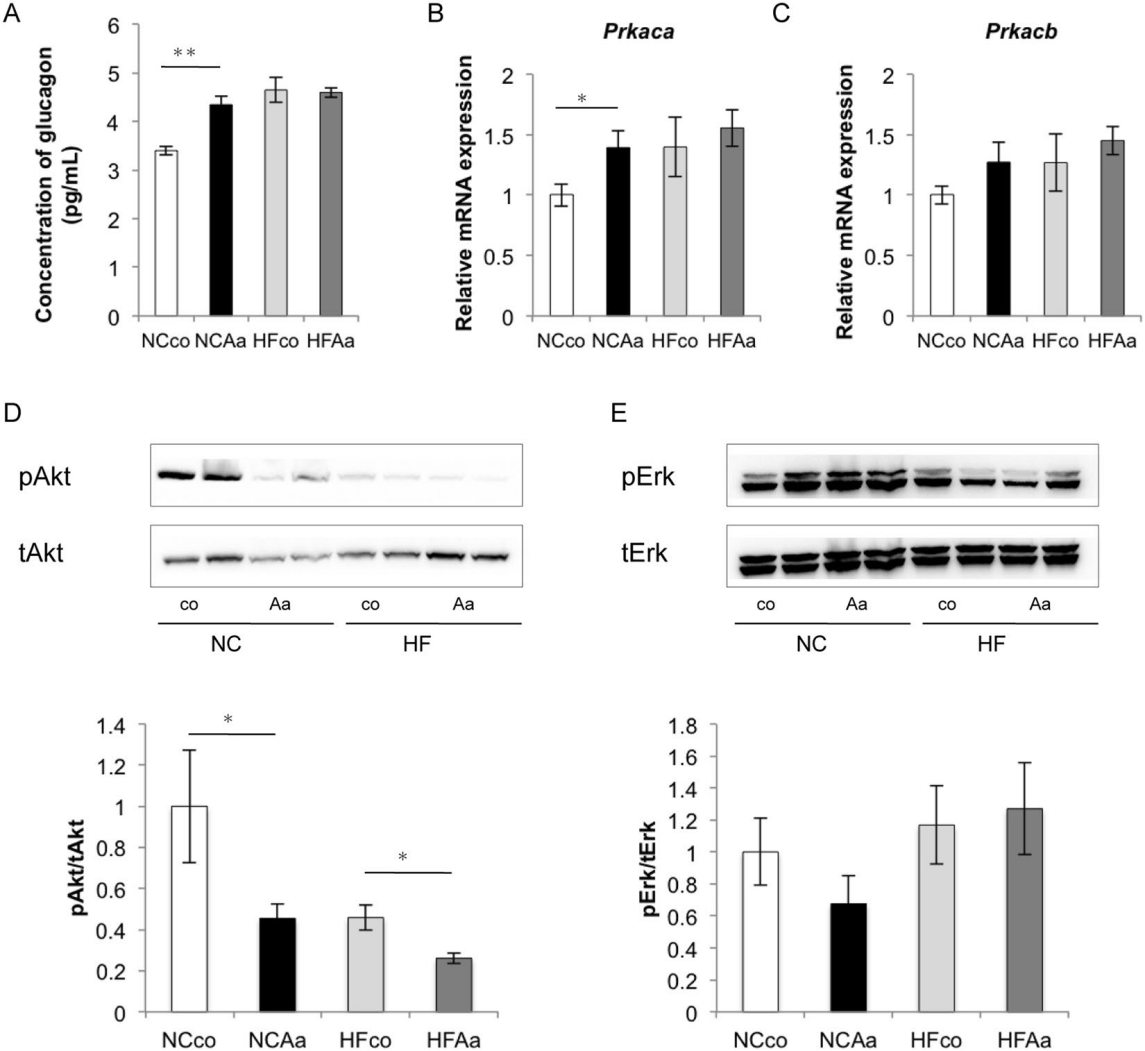
Analysis of glucose metabolism and insulin resistance among NCco, NCAa, HFco and HFAa mice (n = 6–8). (**A**) Concentration of fasting plasma glucagon. Analysis of gene expressions in liver in (**B**) *Prkaca* mRNA expressions, (**C**) *Prkacb* mRNA expressions. (**D**) Total Akt and pAkt expressions, (**E**) total Erk and pErk expressions in liver. *P < 0.05, **P < 0.01.

Based on pathway analysis results, we focused on glucose metabolism in the liver. Fasting plasma glucagon was increased in NCAa compared to NCco mice (Fig. 5A). Protein kinase cAMP-activated catalytic subunit alpha (*Prkaca*) mRNA expression increased significantly after *A*. *actinomycetemcomitans* administration in normally-fed animals (Fig. 5B), although no significant differences could be observed for its beta subunit (*Prkacb*) (Fig. 5C). Furthermore, Akt phosphorylation (pAkt) was significantly lower (by 54%) in the liver of NCAa compared to NCco mice, and in that of HFAa compared to HFco mice (by 43%) (Fig. 5D) without a difference in insulin’s activation of Erk (pErk) (Fig. 5E).

### Evaluation of gut microbiome composition based on 16S rRNA sequences

**Figure 6:**
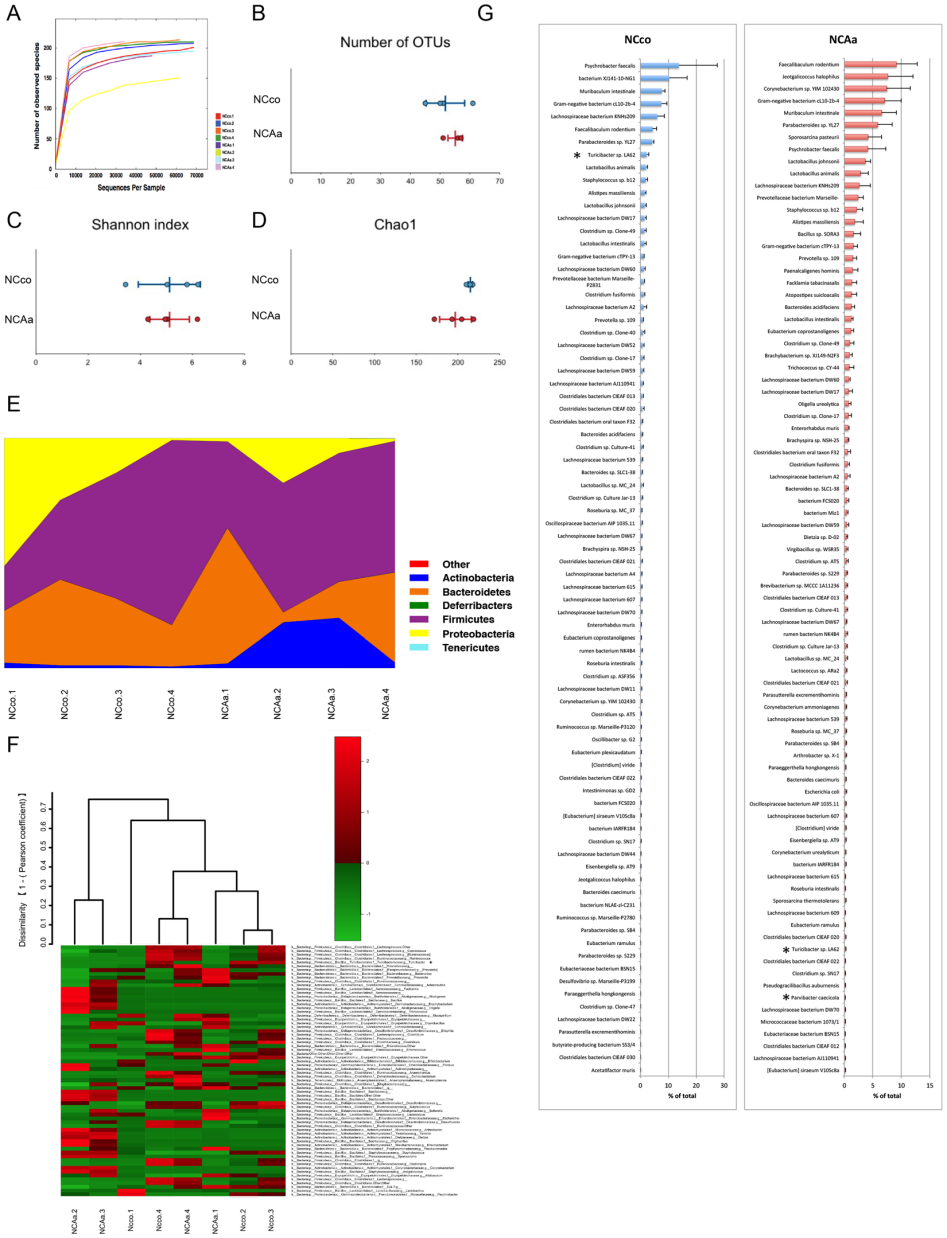
Evaluation of gut microbiome compositions based on 16S rRNA sequences between NCco and NCAa mice (n = 4). (**A**) rarefaction curve, (**B**) number of OTUs, (**C**) Shannon index, (**D**) Chao1 between NCco and NCAa mice. (**E**) Microbial composition at a Phylum level. (**F**) Microbial composition at a Genus level, dendrogram and heatmap constructed based on read abundance. (**G**) Rank distributions of the spices between NCco and NCAa mice (>0.1% relative abundance). The species name or 16S ribosomal RNA database ID in DDBJ is shown. *P < 0.05 between NCco and NCAa mice.

Rarefaction curves indicated that a sufficient number of reads were obtained for 16S rRNA gene analysis (Fig. 6A and Supplementary Fig. S4B). Principal component analysis (PCoA) showed that microbiome composition differed dramatically between mice fed a high-fat or a normal chow diet (Supplementary Fig. S4A). In addition, the number of operational taxonomic units (OTUs), Shannon index, and Chao1 differed significantly between normal chow and high-fat diet feeding mice (P < 0.01), suggesting diversity in gut microbiota were also significantly different between normal chow and high-fat diet feeding mice. To better understand the effect of *A*. *actinomycetemcomitans* administration itself, we evaluated gut microbiota separately according to the diet.

No significant differences in OTUs (Fig. 6B), Shannon index (Fig. 6C), and Chao1 (Fig. 6D) could be detected between NCco and NCAa mice. Although no significant differences in gut microbiota composition between NCco and NCAa mice were observed at a phylum level (Fig. 6E), the genus *Turicibacter* from the phylum Firmicutes was significantly underrepresented in NCAa compared to NCco mice (Fig. 6F). It should be noted that three more species showed significantly different abundance between NCco and NCAa mice (Supplementary Table S2). The most abundant species (>0.1%) in NCco and NCAa mice are presented in Fig. 6G.

A comparison of gut microbiota between HFco and HFAa mice is shown in Supplementary Fig. S4. No significant differences in OTUs, Shannon index, and Chao1 could be detected between HFco and HFAa mice. A same as normal chow diet feeding mice, there were no significant differences in gut microbiota composition between HFco and HFAa mice at a phylum level. The genus *Turicibacter* tended to be underrepresented in HFAa compared to HFco mice.

### Metagenome prediction of gut microbiome

**Figure 7:**
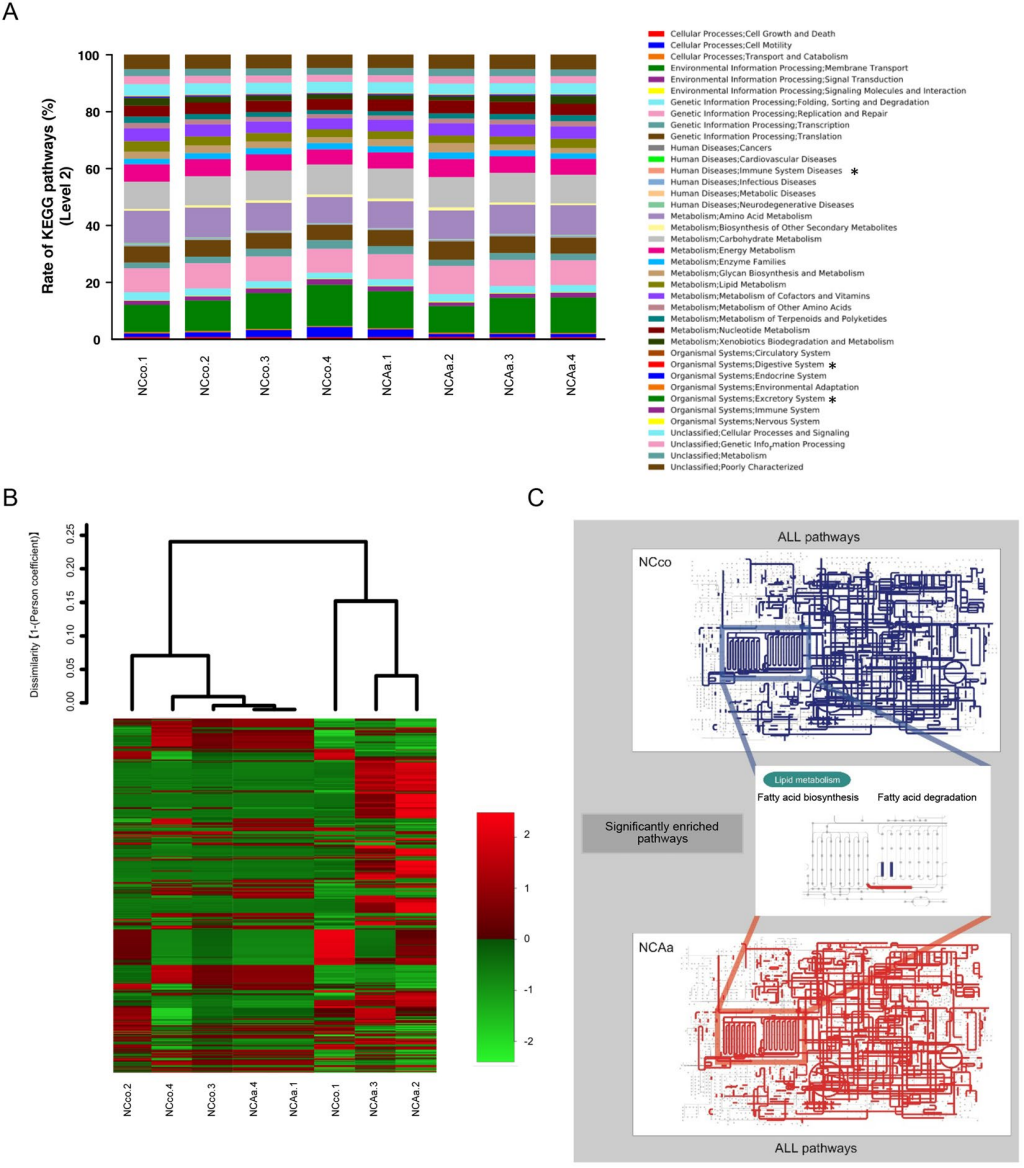
Metagenome prediction between NCco and NCAa mice. (**A**) Metagenome prediction of level-2 subsystem. *P < 0.05 between NCco and NCAa mice. (**B**) Dendrogram and heatmap constructed based on metagenome prediction. (**C**) Predicted KEGG pathways present in any of samples for NCco (upper figure) and NCAa (lower figure). Middle figure shows significantly enriched pathway. Blue: NCco. Red: NCAa.

We performed PICRUSt analysis to predict the relative abundance of gene function in gut microbiome. NCco and NCAa mice presented significantly different functional composition at level 2, particularly with respect to excretory system, digestive system, and immune system diseases (Fig. 7A). Although metagenome prediction was not dramatically different between NCco and NCAa mice (Fig. 7B), 130 functional profiles were predicted to exhibit significant differences between NCco and NCAa mice (Supplementary Table S3). Interestingly, fatty acid biosynthesis was significantly increased, but fatty acid degradation was decreased in NCAa compared to in NCco mice (Fig. 7C).

### Norfloxacin treatment in *Aa*-administered mice

No significant differences in body weight could be detected between NCAa and NCAa mice treated with norfloxacin (NCAaNor), or between HFAa and HFAa mice treated with norfloxacin (HFAaNor) at 12 weeks. Total body fat, visceral fat, and subcutaneous fat volumes tended to be lower in HFAaNor mice compared to HFAa mice at 12 weeks. In addition, norfloxacin treatment partially improved glucose tolerance and insulin resistance in *A*a-administered mice in both dietary groups (Supplementary Fig. S5). Furthermore, norfloxacin treatment could partially supress lipid accumulation in the liver (Supplementary Fig. S6).

## Discussion

The “Multiple hit” hypothesis postulates that multiple insults, such as insulin resistance, LPS, nutritional factors, gut microbiota, and genetic and epigenetic factors, act together to induce NAFLD^17^.

Periodontal diseases are caused predominantly by gram-negative bacteria present in the dental plaque^18^. A only one gram of dental plaque contains more than 10^11^ bacteria^19^. In this study, we measured IgG antibody titers against three major periodontopathic bacteria in NAFLD patients. Detection of oral bacteria in the saliva represents a single-point read-out, whereas the IgG antibody titer is an indicator of chronic infectious status caused by periodontal bacteria. Thus, in this study we focused on IgG antibody titers.

*A*. *actinomycetemcomitans* is frequently detected in severe periodontitis^20^ and is associated with aggressive periodontitis^21^. Among the three kinds of bacteria, only *A*. *actinomycetemcomitans* possess not only endotoxin, but also exotoxin^22^. Leukotoxin, the exotoxin of *A*. *actinomycetemcomitans*, renders this bacterium different from other periodontal pathogens. *F*. *nucleatum* has coaggregration properties, which favour the transport of periodontal bacteria^23^. *P*. *gingivalis* belongs to the “red complex”, a group of bacteria associated with periodontal disease^24^, and is the most common bacterium in periodontal infections.

The association of periodontal disease with NAFLD was first reported by Yoneda *et al*., who suggested that infection with *P*. *gingivalis* might be a risk factor for the development and progression of NAFLD^12^. A recent study using a mouse model showed that administration of *P*. *gingivalis* altered the gut microbiota^25^. In the present study, a positive correlation could be established only between anti-*Aa* IgG antibody titer and visceral body fat, whereas a negative correlation was seen with the L/S ratio. The mean value of the anti-*Aa* IgG antibody titer in 52 NAFLD patients was 9.7. We previously reported that the mean value of the anti-*Aa* IgG antibody titer after periodontal treatment in systemically healthy subjects was 6.9^9^. Therefore, the anti-*Aa* IgG antibody titer appears higher in the 52 NAFLD patients than in systemically healthy subjects. Hence, we focused on *A*. *actinomycetemcomitans* as a risk factor of NAFLD.

KEGG pathway analysis in liver microarrays suggested the induction of metabolic disorders, such as enrichment of glucagon signaling pathway, insulin resistance and adipocytokine signaling pathway, following *A*. *actinomycetemcomitans* administration. What is more, fasting plasma glucagon was increased and *Prkaca* mRNA expression was significantly increased after *A*. *actinomycetemcomitans* administration in animals fed a normal chow diet. Binding of glucagon to its receptor on the hepatocyte plasma membrane leads to activation of adenylyl cyclase, production of the second messenger cyclic AMP, and stimulation of protein kinase A, which phosphorylates protein targets that work in concert to increase hepatic glucose output^26^. Moreover, compared to NCco and HFco animals, and also NCAa and HFAa mice showed lower pAkt/Akt but equal pErk/Erk levels in the liver. This finding is in line with the concept of “selective insulin resistance” by King *et al*.^27^. They reported that elevated levels of glucose, free fatty acids, and inflammatory cytokines caused by diabetes and insulin resistance selectively inhibited insulin’s antiatherogenic actions via the IRS/PI3K/Akt pathway, but not the Grb/Shc/MAPK pathway. In addition, HFAa mice showed enhanced hepatic steatosis by histological analysis.

The gut microbiota has been implicated in NAFLD through its effect on caloric salvage, host energy metabolism, proinflammatory signaling, and via direct hepatotoxicity of bacterial products^28,29^. Furthermore, metagenomic analysis of both microbiome composition and functional capacity correlated with host clinical parameters, energy homeostasis, and proinflammatory signaling in obese and NAFLD patients^30^. There are many reports showing that a high-fat diet dramatically changes gut microbiota in mice^31,32^. Our study also showed remarkable differences in microbiome composition and diversity between normal chow and high-fat diet feeding mice. OTUs of many bacterial species differed significantly between NCco and NCAa mice, and also HFco and HFAa mice, following administration of *A*. *actinomycetemcomitans*. Specifically, the genus *Turicibacter* was underrepresented in *A*. *actinomycetemcomitans* administrated mice. This genus reportedly correlates with production of butyric acid^33^. An increase in butyrate has been associated with improved insulin sensitivity^34^. Therefore, administration of *A*. *actinomycetemcomitans* may affect insulin resistance by altering the gut microbiome and lowering butyrate levels. We also showed that mRNA expression of *Tnfα*, *Il6*, and *Il1β* in the liver did not differ significantly in response to *A*. *actinomycetemcomitans* administration at 6 weeks in mice fed a normal chow diet or a high-fat diet. Therefore, insulin resistance is due to alteration of gut microbiota by *A*. *actinomycetemcomitans* rather than endotoxin-induced inflammation in the liver at 6 weeks.

There are no antibiotics specific to *A*. *actinomycetemcomitans*, so it is impossible to selectively target and disable any function of only *A*. *actinomycetemcomitans*. There are several reports about the effect of antibiotic treatment on glucose metabolism and gut microbiota. Antibiotic treatment improved glucose tolerance and liver lipid accumulation^35^, and also led to changes in gut microbiota^36,37^. Accordingly, we treated *A*a-administered mice with the antibiotic norfloxacin. Norfloxacin treatment improved glucose tolerance, and partially suppressed fat volume and lipid accumulation in the liver. We hypothesised that even if bacterial activity was lost due to antibiotic treatment, norfloxacin could not completely block the effect of *A*. *actinomycetemcomitans* because of the bacterium’s endotoxin/exotoxin.

Intestinal mucosal permeability is greater in NAFLD patients than in healthy subjects^15^. Modulation the gut microbiota is associated with an increased intestinal permeability that precedes the development of metabolic endotoxemia, inflammation, and associated disorders^36^. Gut dysbiosis might cause translocation of bacteria from the gut to the bloodstream^38^. Interestingly, metagenome prediction of the gut microbiota showed increased fatty acid biosynthesis and reduced fatty acid degradation. At level 2, KEGG pathway analysis indicated that oral administration of *A*. *actinomycetemcomitans* altered gut microbiota, causing the upregulation of functional bacterial genes related to immune system disease, in addition, the digestive and excretory systems were capable of processing higher amounts of fatty acid. Accumulating fatty acid in the gut might be transported to the liver via the portal vein, and exacerbate liver steatosis. Moreover, a high concentration of free fatty acids has other effects on the liver, resulting in dyslipidaemia, hyperinsulinaemia, hyperglycaemia, and hepatic insulin resistance^39^. Furthermore, increasing the concentration of fatty acids in the gut may stimulate glucagon secretion by pancreatic alpha cells^40^.

Marked lipid accumulation in the liver was observed in mice fed a high-fat diet, but not in normal chow controls. However, a high-fat diet may in itself alter mRNA expression in the liver. In this study, we intended to evaluate how *A*. *actinomycetemcomitans* administration affected gene expression in the liver. Therefore, we performed microarray analysis to compare normal chow diet control and *Aa*-administered mice. Based on KEGG pathway analysis for differentially expressed genes in the liver of normal chow diet feeding mice, we focused on glucagon signaling pathway, adipocytokine signaling pathway, and insulin resistance. We also confirmed a significant hepatic upregulation of *Ppargc1a*, *Plcb1*, *Acsl1*, and *Ppp2r4* in animals treated with *A*. *actinomycetemcomitans* in high-fat diet feeding.

Administration of *A*. *actinomycetemcomitans* impaired glucose tolerance and insulin resistance in mice fed a high-fat and normal chow diet. There are several reports linking periodontal disease and diabetes^41,42^, which can be explained by the above argument. Our results revealed that mRNA expression of *Tnfα*, *Il6*, *and Il1β* in the liver showed no significant differences between normal chow diet control and normal chow diet with *A*. *actinomycetemcomitans*, or between high-fat diet control and high-fat diet with *A*. *actinomycetemcomitans*. Therefore, insulin resistance is due to the alteration of gut microbiota by *A*. *actinomycetemcomitans* rather than endotoxin-induced inflammation. This study suggests that periodontal disease influences diabetes not only via inflammation, but also by altering gut microbiota.

In conclusion, *A*. *actinomycetemcomitans* might be a risk factor for NAFLD because it alters the gut microbiota. This is the first study to comprehensively evaluate gene expression in the liver and gut microbiota composition following *A*. *actinomycetemcomitans* administration in mice.

## Methods

### Patients

A total of 52 consecutive Japanese patients admitted to Eguchi Hospital and Saga Medical School for the treatment of NAFLD were enrolled in the present study. Patients with evidence of excessive alcohol intake (>20 g/day), other causes of liver diseases (e.g., viral hepatitis, autoimmune liver disease, biliary disease, and hepatocellular carcinoma), or use of antibiotic agents were excluded. NAFLD was diagnosed according to the following criteria: (1) a slight diffuse increase in bright homogeneous echoes in the liver parenchyma, with normal visualisation of the diaphragm and portal and hepatic vein borders, and normal hepatorenal contrast of echogenicity; (2) a diffuse increase in bright echoes in the liver parenchyma, with slightly impaired visualisation of the peripheral portal and hepatic vein borders; and (3) a marked increase in bright echoes at a shallow depth, with deep attenuation and impaired visualisation of the diaphragm and marked vascular blurring^43^, as previously described^44^. BMI was calculated as the body weight in kilograms divided by the square of the height in meters (kg/m^2^). Venous blood samples were taken from all subjects at around 09:00 a.m. following a 12-h overnight fast, and the levels of serum liver enzymes, blood platelet count (×10^4^/μL), total cholesterol (mg/dL), triglycerides (mg/dL), high-density lipoprotein (mg/dL), plasma glucose (mg/dL), and plasma insulin (µg/mL) were determined by standard laboratory techniques. Insulin resistance was calculated by HOMA-IR, using the following formula: HOMA-IR = fasting plasma insulin × fasting plasma glucose/405^45^. Serum from the patients’ venous blood was collected and stored at −80 °C for the analysis of IgG antibody titers.

The study was performed in accordance with the Ethical Guidelines for Clinical Studies (2008 Notification number 415 of the Ministry of Health, Labor, and Welfare). All patients provided informed consent, and the study protocol was approved by the ethics committees of Saga University and Tokyo Medical and Dental University.

### Abdominal computed tomography

Unenhanced spiral acquisition through the liver was obtained during a breath-hold at 5.0-mm collimation, 15.0-mm/rotation table speed (HQ mode, pitch 1:3), 120 kV (p), and auto mA (Light speed QXi; GE Healthcare, Little Chalfont, Buckinghamshire, UK). Images were reconstructed at 10-mm increments. All patients underwent abdominal CT in the morning after a 12-h overnight fast. CT numbers, expressed as Hounsfield units (HU) in a region of interest (ROI) of 40 mm^2^ in the periphery of the liver and the spleen, away from major vessels, were measured at five points in each organ. Mean numbers were used to determine the L/S ratio, an index of fat accumulation in the liver^43,46^. In addition, subcutaneous fat area (cm^2^) and visceral fat area (cm^2^) were measured at the umbilical level and calculated using FatScan software (N2 System, Osaka, Japan)^47^.

### Cultivation of *A.actinomycetemcomitans*

*A*. *actinomycetemcomitans* ATCC 43718 was inoculated in ATCC Medium 44 (Brain Heart Infusion Broth) and cultured anaerobically (AnaeroPack^R^-Anaero for Susceptibility, Mitsubishi Gas Chemical Company Inc., Tokyo, Japan) at 37 °C for 24 h.

### Serum IgG titer measurement

Specific serum IgG titers were measured by enzyme-linked immunosorbent assay (ELISA) as described previously^9,48^, using sonicated whole cell extracts of each periodontal bacteria. Briefly, 96-well microplates (EIA plate; Costar, Corning, NY, USA) were coated with sonicated *A*. *actinomycetemcomitans*, *F*. *nucleatum*, or *P*. *gingivalis* at 10 μg/mL in carbonate buffer and incubated for 2 h at 37 °C. After blocking with 2% bovine serum albumin in carbonate buffer, plates were washed with phosphate-buffered saline (PBS)-Tween (1×PBS, 0.05% Tween 20, pH 7.2). Serially diluted reference positive control serum (2^5^–2^15^, 200 µL per well) and single diluted patient serum (2^10^, 200 µL per well) were added into each well, after which the plates were incubated for 1 h at 37 °C and washed again. Subsequently, 200 µL per well of alkaline phosphatase-conjugated goat anti-human IgG (Sigma, St. Louis, MO, USA) was added. Following incubation, plates were washed and developed with phosphatase substrate (Sigma). Optical density at 450 nm was read using a microplate reader (SoftMAX, Molecular Devices, Sunnyvale, CA, USA). Antibody titers were calculated according to the method of Wang *et al*.^48^.

### Animals

C57BL/6J mice (8-weeks-old; Sankyo Laboratory, Tokyo, Japan) were allowed free access to water and food throughout the experimental period. Mice were randomly divided into four groups: NCco, NCAa, HFco, and HFAa. HFAa and HFco mice were fed High-fat diet 32 (Japan CLEA, Tokyo, Japan); this feed contains 506.8 kcal/100 g (57.5% from fat, 19.7% from protein, and 22.8% from carbohydrate). A total of 10^8 ^*A*. *actinomycetemcomitans* cells suspended in 100 μL of physiological saline solution were given to NCAa and HFAa mice via a feeding needle. The suspension was given 6 times per week for 6 weeks. NCco and HFco mice were given saline only. To block the effect of *A*. *actinomycetemcomitans*, norfloxacin was administered (3 g/mouse) 6 times per week for 6 weeks.

GTT and ITT were performed as previously described^49^. Briefly, after fasting for 6 h, mice were fed glucose by oral gavage (1 g/kg) or were given an intraperitoneal injection of insulin (1 U/kg) (Humalin R; Eli Lilly and Company, Indianapolis, IN, USA). Glucose concentration was determined with a glucose meter (ACCU-CHEK ST Meter; Roche, Basel, Switzerland). The area under the curve (AUC) (0–120 min) was calculated for each group of mice. Plasma glucagon was measured by the Glucagon ELISA kit (Mercodia, Uppsala, Sweden) after fasting for 6 h.

To evaluate insulin signaling, we fasted the mice for 6 h, injected them with insulin at 1.5 U/kg, and waited 20 min before sacrificing them and freezing the liver at −80 °C.

All experiments with mice were conducted according to the Guidelines for Proper Conduct of Animal Experiments, Science Council of Japan, and protocols were approved by the Animal Care Committee of the Experimental Animal Center at Tokyo Medical and Dental University (0170225 A).

### Evaluation of visceral and subcutaneous fat ***in vivo***

Micro-CT imaging was performed with a micro-CT unit (RmCT2; Rigaku Corporation, Tokyo, Japan) with FOV60 for 2 min, on mice anesthetised with isoflurane (induction 2%, maintenance 1.5%). The CT images were visualised and analysed using CTAtlas Metabolic Analysis (ver. 2.03) software (Rigaku Corporation). The fat tissue HU value was adjusted to between −350.0 and −145.0 in accordance with the manufacturer’s instructions. Measurement of body fat volume was limited to the abdominal region, with the initial point set at the diaphragm. Body fat was then divided into visceral fat and subcutaneous fat along the ribs^50^.

### RNA preparation and quantitative real-time PCR

Total RNA was extracted from liver tissue using the NucleoSpin® RNA kit (TaKaRa Bio, Shiga, Japan) and quantified by measuring absorbance at 260 and 280 nm. Next, 500 ng total RNA was reverse-transcribed to cDNA using the PrimeScript^TM^ RT Master Mix (TaKaRa Bio), and real-time PCR was performed using the Thermal Cycler Dice® Real Time System II (TaKaRa Bio). PCR mixtures were prepared by SYBR® Premix Ex Taq™ II (TaKaRa Bio), and PCR conditions were determined according to the manufacturer’s protocol. Gene expression levels were normalised to those of the reference gene, *36b4*. PCR primers used in the study are listed in Supplementary Table S4.

### Liver lysate preparation and western blotting

Lysates from frozen livers were extracted with RIPA buffer (150 mM NaCl, 50 mM Tris, pH 7.6, 1% Triton X-100, 0.5% sodium deoxycholate, 0.1% SDS, supplemented with protease and phosphatase inhibitors), cleared, and used for western blotting^51^. Antibodies against Erk, pErk, Akt, and pAkt were purchased from Cell Signaling Technology (Danvers, MA, USA); goat anti-rabbit IgG was purchased from Santa Cruz Biotechnology Inc. (Santa Cruz, CA, USA).

### Liver histological analysis

Liver samples were fixed with 4% paraformaldehyde in PBS for 24 h and embedded in paraffin. Next, 5-μm-thick sections were processed with haematoxylin and eosin (HE); the lipid area was quantified using ImageJ software^52^.

### Microarray and data analysis

Total RNA was extracted from the liver of NCco and NCAa mice, and run on an Agilent 2100 Bioanalyzer (Agilent Technologies, Santa Clara, CA, USA) to check sample quality. The Agilent Low Input Quick Amp Labeling kit was used to generate cRNA with a sample input of 200 ng total RNA for single-color microarray (Cy3) analysis according to the manufacturer’s instructions. Then, cRNA was analysed by hybridisation onto an Agilent SurePrint G3 Unrestricted Gene Expression 8 × 60 K Microarray. Fluorescence signal from the hybridised microarrays was detected using the Agilent Microarray Scanner System. Raw microarray data were extracted using Feature Extraction Software (ver. 11.0.1.1; Agilent Technologies).

Microarray data were 75% tile-normalised and log_2_-transformed according to the manufacturer’s recommendation^53^ using R software (ver. 3.3.2). The Limma Bioconductor package (ver. 3.30.4)^54^ was used to identify DEGs. Statistical significance was measured by Benjamin and Hochberg’s false discovery rate (FDR) to account for multiple testing. DEGs were selected according to FDR < 0.1 and |fold-change| > 1.5. Overrepresentation enrichment analyses for DEGs were performed with the WEB-based Gene SeT AnaLysis Toolkit (http://www.webgestalt.org)^55^ using GO and KEGG pathway databases.

### 16S rRNA gene sequencing and Illumina sequence data processing

DNA was extracted from mouse faeces using the NucleoSpin Microbial DNA kit (Machery Nagel, Düren, Germany) and purified with the Agencourt AMPure XP PCR system (Beckman Coulter, Beverly, MA, USA). A multiplexed amplicon library covering the 16S rDNA (V3-V4) region was generated from extracted DNA samples using the 16S (V3-V4) Metagenomic Library Construction Kit for NGS (TaKaRa Bio) following the manufacturer’s protocol and purified again with the AMPure XP system. The Illumina Miseq platform (Illumina Inc., San Diego, CA, USA) was used to generate 250-bp paired-end sequences. The obtained sequence data are available in the DNA Data Bank of Japan (http://www.ddbj.nig.ac.jp/) under accession number DRA005604 and DRA006060. A total of 2,031,033 sequence reads were generated, corresponding to an average of 126939.6 (range, 90,723–180,451) reads per sample. Noise, low quality sequences, pyrosequencing errors, and chimeras were removed from the data set. The preprocessed reads were clustered into OTUs at 97% identity using the CD-HIT-OTU pipeline (http://weizhongli-lab.org/cd-hit-otu/, ver. 0.01)^56^.

### Taxonomic assignment and metagenome prediction

OTUs were processed and analysed with the Quantitative Insights into Microbial Ecology (QIIME, ver. 1.8) software package^57^. Taxonomic classification of the sequences at the phylum or genus level was determined using the RDP classifier (ver. 2.2) with default parameters against the GreenGenes database (ver.:gg_13_8). Taxonomic assignment was refined at the species level based on the 16S rRNA database (DNA Data Bank of Japan, as of July 18, 2017) using BLASTN. Hits with E values ≤ 1e-5 were considered significant. Alpha-diversity indices were estimated from the number of observed OTUs, and the Shannon diversity index^58^ was used as a measure of species richness and evenness. To calculate species richness based on OTUs, rarefaction curves were generated using QIIME with default parameters. At the same time, the PICRUSt (ver. 1.0.0) bioinformatics software package^59^ was employed to generate metabolic predictions based on closed OTUs at a 97% similarity level. Samples were normalised by randomly resampling the sequences to 10,000 reads per sample using the Seqtk application (https://github.com/lh3/seqtk). The analysed OTUs were normalized to 16S rRNA copy number. Functional composition of the data was predicted based on the KEGG database^60^. Dendrograms with heatmaps were visualised using R software (ver. 3.3.2). Dissimilarity values (1 – Pearson correlation) were clustered using average linkage methods.

### Statistical analysis

Data distribution was assessed using the Shapiro-Wilk test. Correlation between anti-periodontal bacteria IgG antibody titers and clinical/biochemical parameters was evaluated by Spearman’s rank correlation coefficient. In animal experiments, Student’s *t*-test was applied to compare two groups. One-way analysis of variance, followed by a *t*-test with Bonferroni connection, was performed for multiple groups’ comparisons using SPSS 22.0 software (SPSS Inc., Chicago, IL, USA). A value of P < 0.05 was considered statistically significant.

## Electronic supplementary material

Supplementary Information

Supplementary Dataset S1, Supplementary Dataset S2, Supplementary Dataset S3.
