# Author Correction: Periodontal pathogenic bacteria, *Aggregatibacter actinomycetemcomitans* affect non-alcoholic fatty liver disease by altering gut microbiota and glucose metabolism

**DOI:** 10.1038/s41598-018-23000-6

**Published:** 2018-03-12

**Authors:** Rina Komazaki, Sayaka Katagiri, Hirokazu Takahashi, Shogo Maekawa, Takahiko Shiba, Yasuo Takeuchi, Yoichiro Kitajima, Anri Ohtsu, Sayuri Udagawa, Naoki Sasaki, Kazuki Watanabe, Noriko Sato, Naoyuki Miyasaka, Yuichiro Eguchi, Keizo Anzai, Yuichi Izumi

**Affiliations:** 1 Department of Periodontology, Graduate School of Medical and Dental Sciences, Tokyo Medical and Dental University (TMDU), Tokyo, Japan; 2 Division of Metabolism and Endocrinology, Faculty of Medicine, Saga University, Saga, Japan; 3 Eguchi Hospital, Ogi, Saga Japan; 4 Department of Molecular Epidemiology, Medical Research Institute, Tokyo Medical and Dental University (TMDU), Tokyo, Japan; 5 Department of Comprehensive Reproductive Medicine, Tokyo Medical and Dental University (TMDU), Tokyo, Japan; 6 Liver Center, Saga University Hospital, Saga, Japan

Correction to: *Scientific Reports* 10.1038/s41598-017-14260-9, published online 24 October 2017

This Article contains errors in Table 1, where incorrect values for ‘Fasting plasma glucose (mg/dL)’ and ‘L/S ratio’ are given. The correct Table 1 appears below.

**Table 1:** Characteristics of the patients with NAFLD.

Variables	All patients	Males	Females
	(n = 52)	(n = 27)	(n = 25)
Age (years)	55 ± 13.8	48 ± 14.6	63 ± 7.5
Weight (kg)	68 ± 12.0	75 ± 11.0	59 ± 6.2
BMI (kg/m^2^)	26 ± 2.8	27 ± 3.2	26 ± 2.2
Blood platelet count (×10^4^/μL)	22 ± 5.5	22 ± 5.3	22 ± 4.7
AST (IU/L)	26 ± 9.1	28 ± 9.5	23 ± 8.1
ALT (IU/L)	30 ± 18.2	37 ± 20.0	22 ± 12.2
ALP (IU/L)	243 ± 69.8	206 ± 63.2	284 ± 52.1
γ-GTP (IU/L)	57 ± 74.4	80 ± 95.8	31 ± 18.6
Fasting plasma glucose (mg/dL)	105 ± 14	106 ± 16	104 ± 11
HOMA-IR	2.1 ± 1.5	1.8 ± 1.1	2.4 ± 1.9
Total cholesterol (mg/dL)	219 ± 48	227 ± 46	218 ± 25
High density lipoprotein (mg/dL)	57 ± 14	54 ± 14	62 ± 12
Triglyceride (mg/dL)	145 ± 100	165 ± 128	124 ± 58
L/S ratio	1.12 ± 0.21	1.07 ± 0.26	1.17 ± 0.15

Data are shown as mean ± SD.

In addition, this Article contains a typographical error in the Methods section, under the subheading ‘Statistical analysis’ where,

“Bonferroni connection”

should read:

“Bonferroni correction”

