# Supplementary material for: Periodontal pathogenic bacteria, Aggregatibacter actinomycetemcomitans affect non-alcoholic fatty liver disease by altering gut microbiota and glucose metabolism
Source: Sci Rep. 2017 Oct 24;7:13950. doi: 10.1038/s41598-017-14260-9 (PMC5655179; doi:10.1038/s41598-017-14260-9)
Supplement: Supplementary file 1 — Supplementary Information [file 41598_2017_14260_MOESM1_ESM.pdf]

Supplementary Information

Periodontal pathogenic bacteria, *Aggregatibacter actinomycetemcomitans* affect non-alcoholic fatty liver disease by altering gut microbiota and glucose metabolism

Rina Komazaki, Sayaka Katagiri, Hirokazu Takahashi, Shogo Maekawa, Takahiko Shiba, Yasuo Takeuchi, Yoichiro Kitajima, Anri Ohtsu, Sayuri Udagawa, Naoki Sasaki, Kazuki Watanabe, Noriko Sato, Naoyuki Miyasaka, Yuichiro Eguchi, Keizo Anzai, Yuichi Izumi

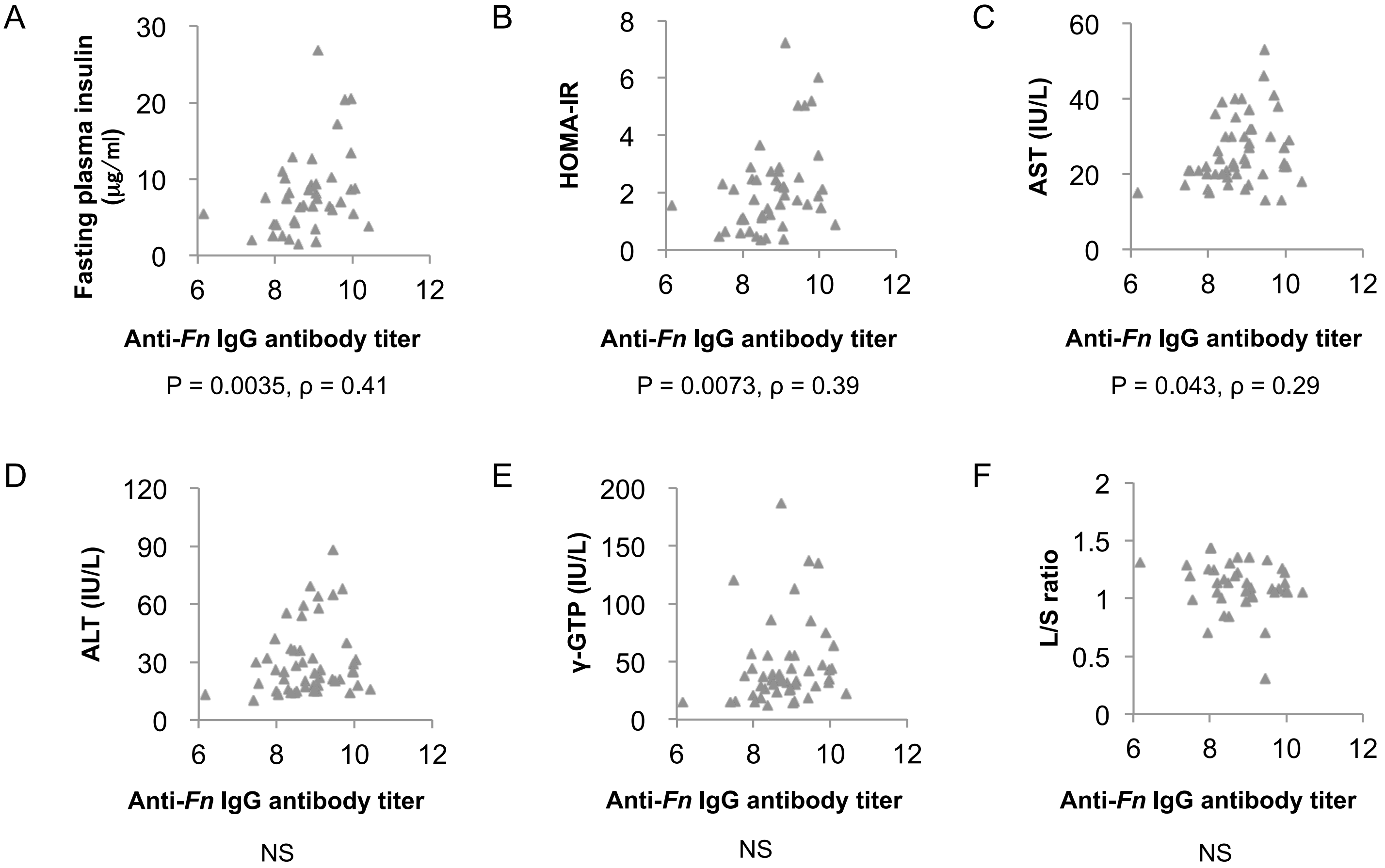

Supplementary Figure S1: Correlations between IgG antibody titer to *Fusobacterium nucleatum* and clinical/ biochemical parameters in NAFLD patients (n=52).
Correlation between anti-*Fn* IgG antibody titer and (A) fasting plasma insulin, (B) HOMA-IR, (C) AST, (D) ALT, (e) γ-GTP, (F) L/S ratio.

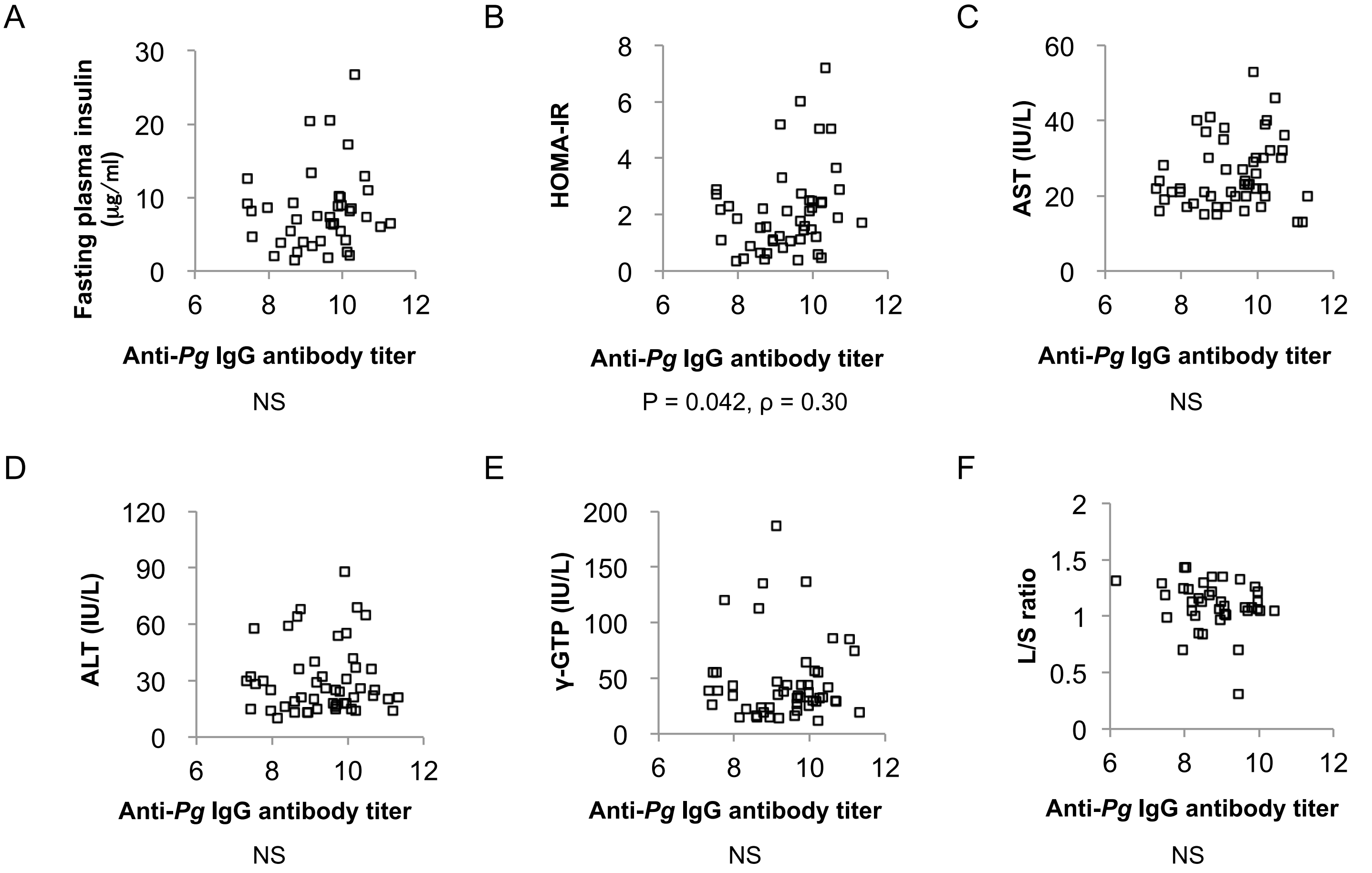

Supplementary Figure S2: Correlations between IgG antibody titer to *Porphyromonas gingivalis* and clinical/ biochemical parameters in NAFLD patients (n=52).
Correlation between anti-*Pg* IgG antibody titer and (A) fasting plasma insulin, (B) HOMA-IR, (C) AST, (D) ALT, (e) γ-GTP, (F) L/S ratio.

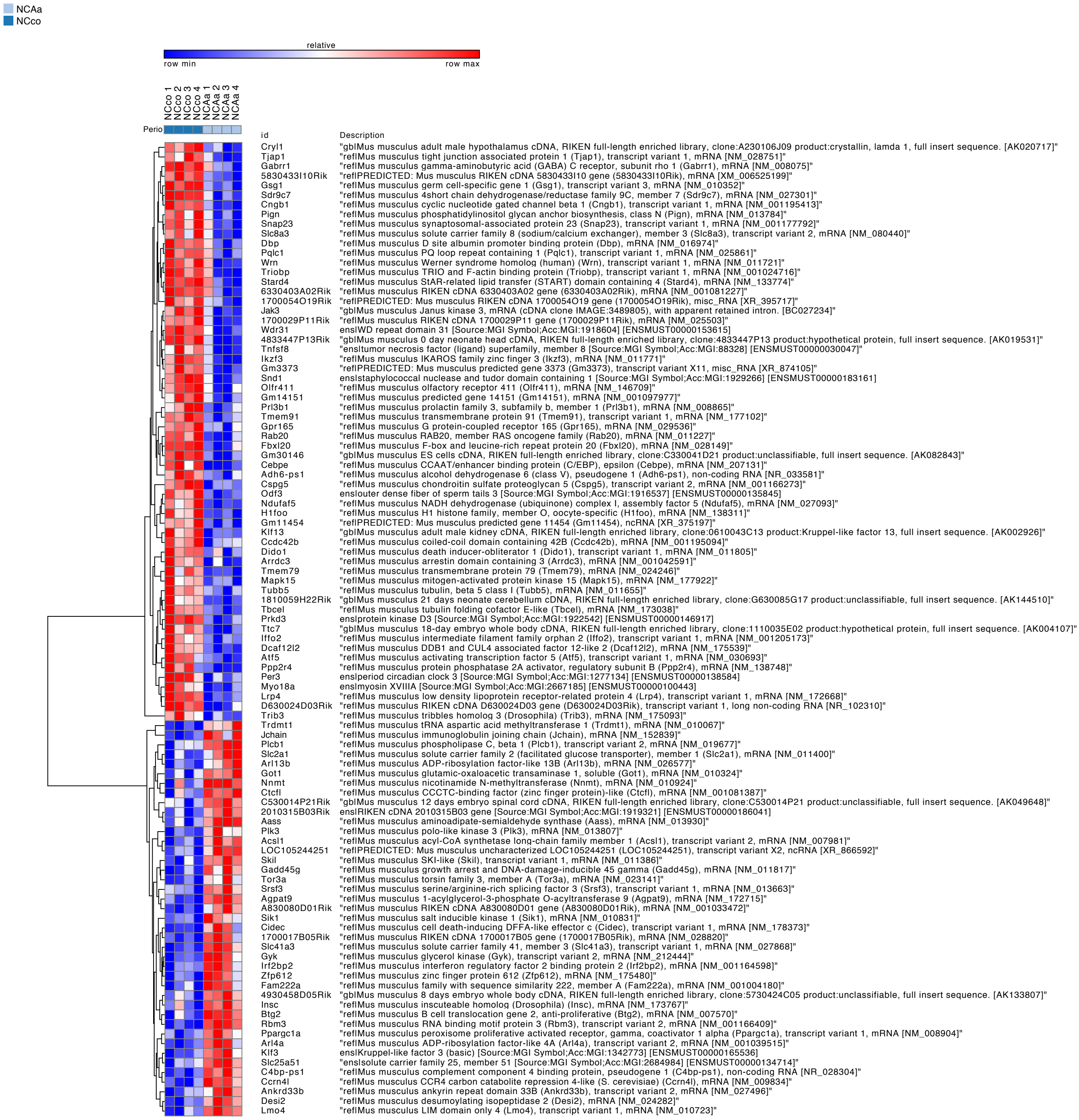

Supplementary Figure S3: Heatmap for DEGs with higher expression.

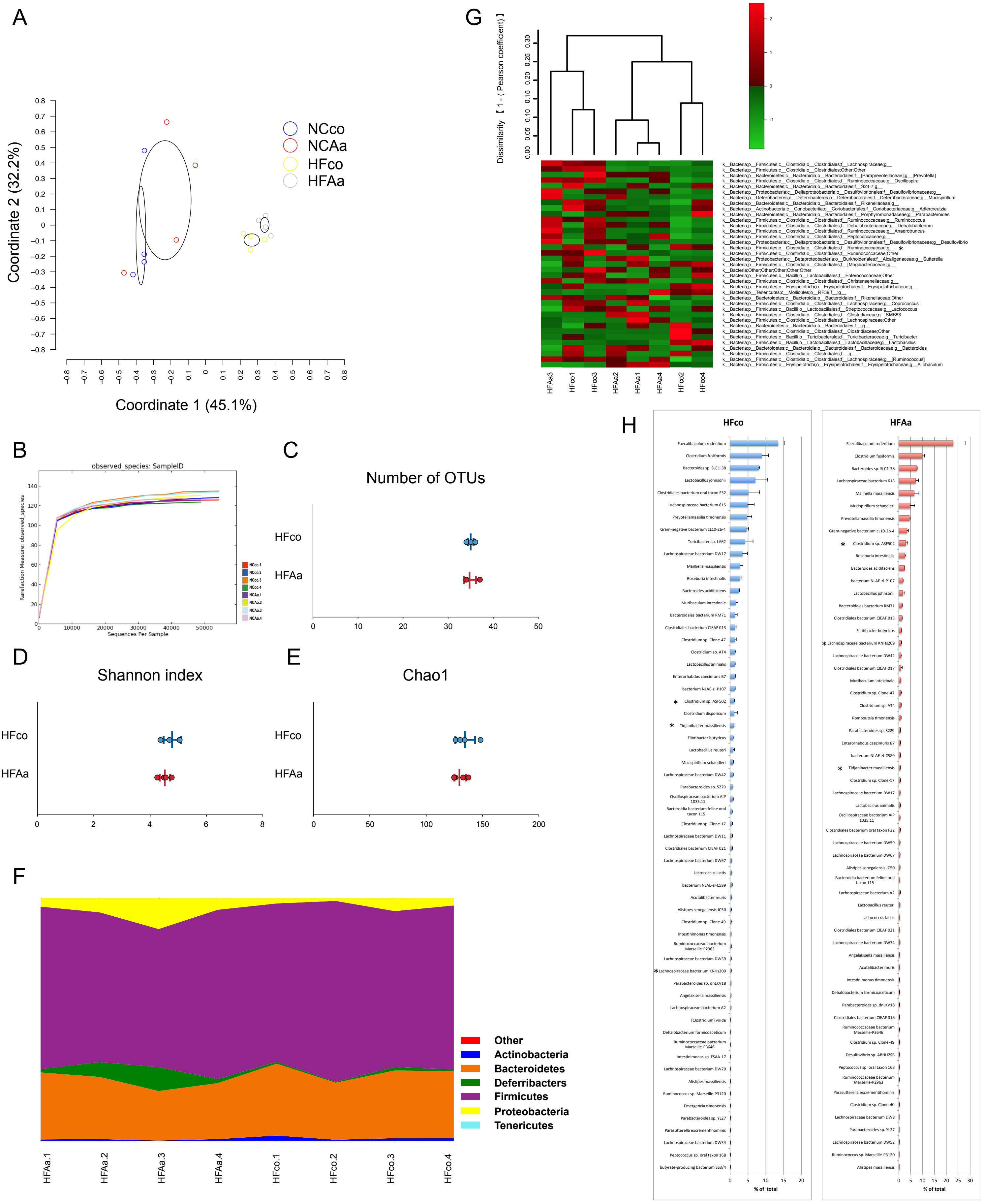

Supplementary Figure S4: Evaluation of gut microbiome compositions based on 16S rRNA sequences between HFco and HFAa mice (n= 4). (A) PCoA analysis among NCco, NCAa, HFco and HFAa mice. (B) rarefaction curve, (C) number of OUT, (D) Shannon index, (E) Chao1 between HFco and HFAa mice. (F) Microbial composition at a Phylum level. (G) Microbial composition at a Genus level, dendrogram and heatmap constructed based on read abundance. (H) Rank distributions of the spices between HFco and HFAa mice (>0.1% relative abundance). The species name or 16S ribosomal RNA database ID in DDBJ is shown. *: P < 0.05 between HFco and HFAa mice.

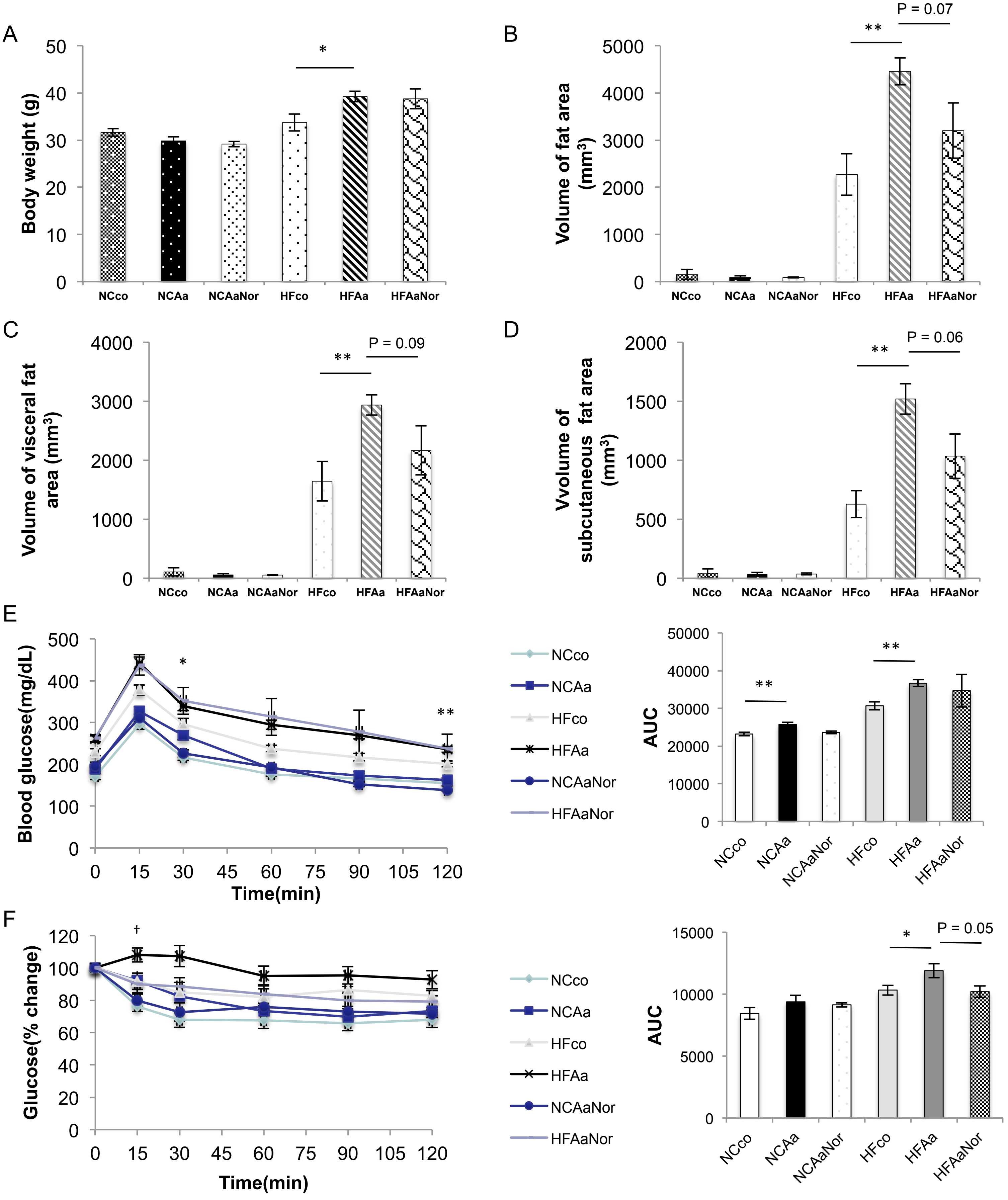

Supplementary Figure S5: Comparison of body weight, body fat, glucose tolerance and insulin resistance among NCco, NCAa, NCAaNor, HFco, HFAa and HFAaNor mice.

(A)Body weight. (B) The volume of fat area, (C) the volume of visceral fat area and (D) the volume of subcutaneous fat area evaluated by Micro-CT imaging at 12 weeks (n=5). *: $P < 0.05$, **: $P < 0.01$.

(E) OGTT (1 g/ kg) and (F) ITT (1 U/ kg) performed 6 h fasting at 6 weeks.*: $P < 0.05$, **: $P < 0.01$ NCAa vs NCAaNor, †: $P < 0.05$ HFAa vs HFAaNor.

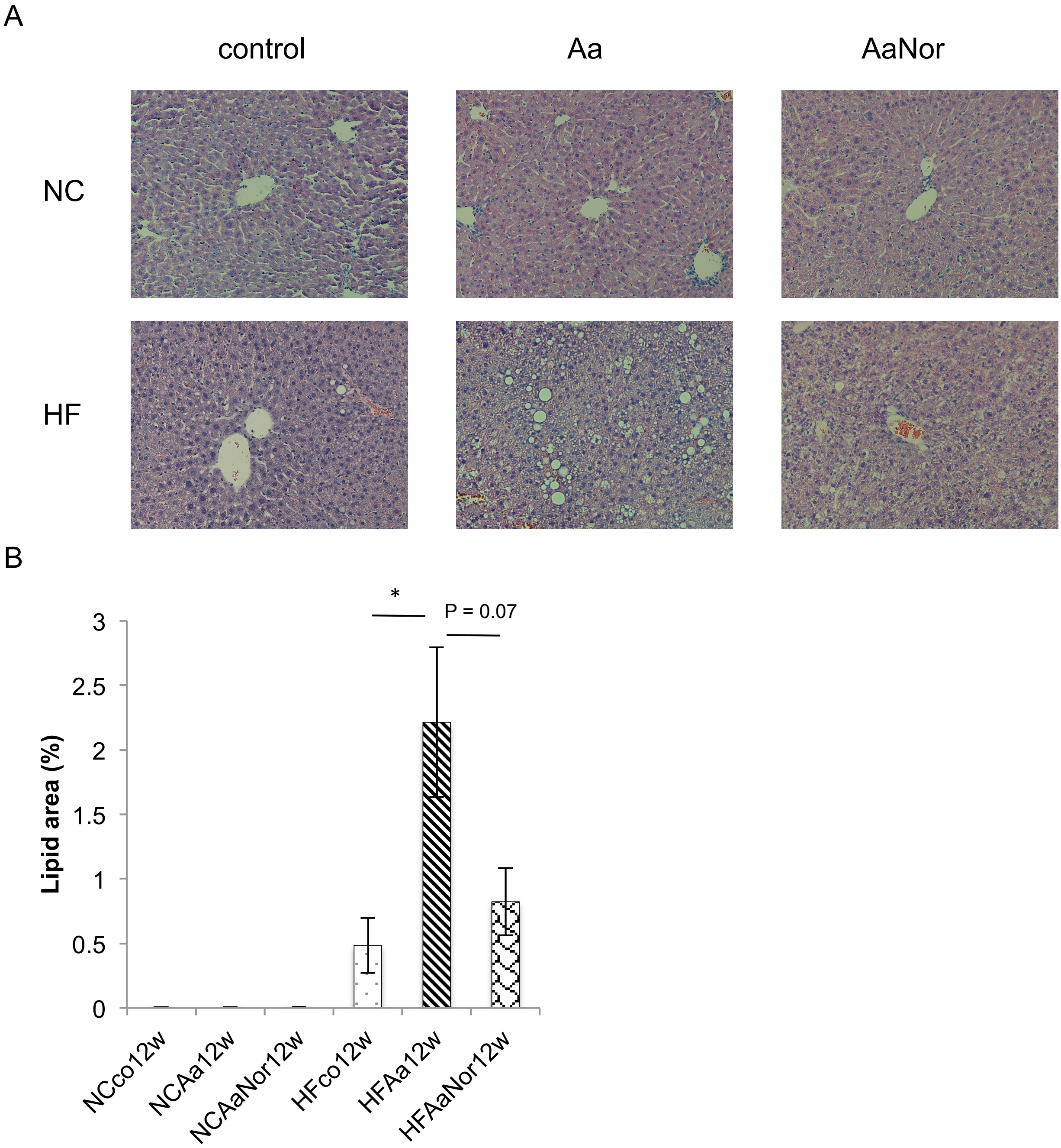

Supplementary Figure S6: Evaluation of liver steatosis among NCco, NCAa, NCAaNor, HFcp, HFAa and HFAaNor mice (n=5).
(A) HE staining of liver tissue from NCco, NCAa, NCAaNor, HFco, HFAa and HFAaNor mice at 12 weeks (row magnification ×200, Black bar = 100 μm), and (B) lipid area (%).

Supplementary Table S4: Primers used for quantitative PCR analysis.

Gene	Primers	
	Sense (5'-3')	Anti-sense (5'-3')
Acc1	ACACCATGTTGGGAGTTGTG	GCTGTTCCTCAGGCTCACAT
Glck	TATGAAGACCGCCAATGTGA	TTTCCGCCAATGATCTTTTC
Il1β	CTGTGACTCATGGGATGATGATG	CGGAGCCTGTAGTGCAGTTG
Tnfα	ACGGCATGGATCTCAAAGAC	AGATAGCAAATCGGCTGACG
Il6	TAGTCCTTCCTACCCCAATTTCC	TTGGTCCTTAGCCACTCCTTC
Ppargc1a	ATGACCCTCCTCACACCAAACCCACAG	CTTGAGCATGTTGCGACTGCGGTTGTG
Slc2a1	CAGTTCGGCTATAACACTGGTG	GCCCCCGACAGAGAAGATG
Plcb1	GGGGTACCCCAAATGCTTGTCTGGCCTCC	GCTCTAGAGCCTGGTGAACTATATTCAGCC
Sik1	TGGACGTCTGGAGCCTCGGT	AGAGTGGGGTCGGCCTGCAT
Ppp3cc	ATGCCACCCCGAAAAGAGG	CATGGTCGGTCCTTCTTGACG
Acsl1	TGCCAGAGCTGATTGACATTC	GGCATACCAGAAGGTGGTGAG
Ppp2r4	TGGATTCGACTTAGACTTGACCT	GCGGTGTCATAATGTCTCTCAG
Prkaca	AGATCGTCCTGACCTTTGAGT	GGCAAAACCGAAGTCTGTCAC
Prkacb	CTCGGGACGGGTTCCTTTG	AGGGACGTATTCCATAACCATGT
36b4	GCTCCAAGCAGATGCAGCA	CCGGATGTGAGGCAGCAG
